# Buffalo Obstetrical Society

**Published:** 1890-06

**Authors:** Wm. C. Krauss

**Affiliations:** Secretary


### BUFFALO OBSTETRICAL SOCIETY.

Reported by WM. C. KRAUSS, M. D., Secretary.

REGULAR meeting held Tuesday evening, April 29th.

The essayist of the evening, Dr. W. S. Tremaine, spoke at some length upon Puerperal Hysterectomy, giving a brief history of the operation from its conception down to the present time. The technique of the operation was carefully and fully described, also the modifications, viz.: The improved Cesarean section or Sänger's operation, Porro's operation, and Thomas's operation or lapara-elytrotomy. Porro's operation is simply Cesarean section plus hysterectomy, the object being to prevent future conceptions. The statistics bearing upon these different operations, the treatment of the pedicle, drainage, etc., were all carefully taken up and presented with much completeness and precision.

### DISCUSSION.

Dr. R. L. BANTA.—I have had but two cases where it was necessary to do craniotomy, and two Cesarean sections. In one case I did craniotomy and the second time had rupture of the lower segment of the womb. Had a Porro been done the case might have been saved. I agree with the essayist that every obstetrician should know how to do a Cesarean section, but in all cases should call in an expert operator.

Dr. W. W. POTTER.—The question of statistics is of considerable interest. The following from Dr. Harris, published in Volume II., Transactions of the American Association of Obstetricians and Gynecologists, shows the relative status of the Porro-Cesarean and Sänger-Cesarean operations:

| | | | |
|---|---|---|---|
| Porro operations in 15 countries, | 267; | women lost, 121; | children, dead or dying, 49. |
| Sänger " 11 " | 196; | " " 48; | " " " 17 |
| Porro mortality, | 1 in $2\frac{1}{5}$. | | |
| Sänger " | 1 in $4\frac{1}{12}$. | | |
| Porro operations of Italy, | 92; | women lost, 44; | children, dead or dying, 14 |
| Sänger " of Germany, | 92; | " " 13; | " " " " 8 |
| Porro operations of Vienna, | 37; | women lost, 18; | women saved, 56 per cent. |
| Sänger " " " | 14; | " " 1; | " " $92\frac{6}{7}$ " |
| Porro operations of Austria, | 61; | women lost, 18; | women saved, $70\frac{1}{2}$ per cent. |
| Sänger " " " | 32; | " " 6; | " " $81\frac{1}{4}$ " |

| | | | | |
|---|---|---|---|---|
| Porro operations of Germany, | 41; | women lost, 21; | women saved, | $48\frac{3}{4}$ per cent. |
| Sänger " " " | 92; | " " 13; | " " | $85\frac{1}{2}$ " |
| Porro operations of the United States, | 9; | women lost, 7; | women saved, | $22\frac{2}{9}$ per cent. |
| Sänger " " " | 33; | " " 18; | " " | $45\frac{15}{33}$ " |

| | | | | |
|---|---|---|---|---|
| Sänger operations in Dresden | under 6 operators | 31; | women saved, 27 | Loss 8 women, or 1 in $9\frac{1}{8}$ |
| " " " Leipzig | " 6 " | 20; | " " 18 | |
| " " " Berlin | " 5 " | 8; | " " 7 | |
| " " " Vienna | " 6 " | 14; | " " 13 | |
| | | 73 | 65 | |

In a recent letter to Dr. Wathen, Dr. Harris states that "Cesarean statistics are changing daily; about six Sänger operations are performed on an average monthly, and case No. 208 is now in her tenth day. If she recovers, it will make 16 out of 34 for the N. S., and 157 out of 204 for the world, in twelve countries, with 187 children also saved. The Porro operations are 269, with 147 women saved and 229 children delivered alive." Much can be said about the Sänger and Porro operations, the others are of secondary importance. Porro's is conceded to be the easier of the two, as considerable expertness is necessary to do a Sänger. It is best, however, to leave the question of choice to a skilled operator in the presence of his patient.

Dr. P. W. Van Peyma.—I was once called by a midwife to see a case, and found the head low down, with one arm engaged. Gave chloroform, turned and delivered the child. On making an examination found that a rupture of the lower segment of the uterus and upper segment of the vagina had taken place. On the seventh day patient was up and around. Once did a post mortem Cesarean section and found rupture of the uterus, probably due to large doses of ergot administered by the midwife. Some operators decline to do craniotomy for theological reasons.

Dr. C. C. Frederick.—Why not in Sänger's operation remove the tubes and ovaries; it will unsex the woman and prevent future conceptions? Physicians would rather resort to craniotomy than call in an expert to do a Cesarean section.

Dr. Tremaine.—The reason that Dr. Banta has had so few cases for Cesarean section, is because his cases have been of the robust kind. In the other classes, obstetrics becomes more difficult every day. Rupture of the uterus always calls for Cesarean section. In regard to removing the tubes and ovaries in Sänger's operation, time is the important question, because the woman is debilitated by long labor, hemorrhage, shock on opening the abdomen, and the like.

